# Distress Tolerance as a Moderator of Affective Forecasting Effects

**DOI:** 10.1007/s42761-025-00303-2

**Published:** 2025-05-06

**Authors:** Roscoe C. Garner, Evan M. Kleiman

**Affiliations:** Department of Psychology, Rutgers, The State University of New Jersey, New Brunswick, NJ USA

**Keywords:** Distress tolerance, Affective forecasting, Ecological momentary assessment, Negative affect

## Abstract

Previous research has used real-time methods like ecological momentary assessment (EMA) to examine affective forecasting (predictions made regarding experience of affect and how this influences decision making, level of functioning, etc.), but has not specifically examined predictors of what determines how strongly daily forecasts of a day are associated with negative emotion experienced later in the day. The aim of this study was to examine how experience of distress tolerance would moderate daily-level affective forecasting effects. Our hypothesis stated that having poorer distress tolerance would mean that worse predictions of how good a day would be, would be more strongly associated with negative emotion later in the day. Analyses in a large sample of undergraduates (*N* = 411 drawn from a sample total sample of *N* = 675) supported this hypothesis. Future research should look to further explore distress tolerance as a moderator of affective forecasting, specifically within larger community samples.

Affective forecasting refers to predictions that individuals make regarding future affective experiencing, and thus how this can influence decision making, level of functioning, and goal-directed behavior. This can be broken down into four key components: predictions about the valence of one’s feelings in the future, specific emotions the individual will be experiencing, intensity of the emotions, and the duration of emotions (Wilson & Gilbert, 2003). An example of this can be students who are struggling may fall into all-or-nothing thinking, believing that nothing in their life will improve (Gilbert, 1998). Previous research regarding affective forecasting and ecological momentary assessment (EMA) has been conducted. For example, one study examined how affective forecasting predictions compared to using ecological momentary assessment (EMA) ratings for individuals with schizophrenia, finding that participants expected more emotionally charged weeks than actually experienced. (Brenner & Ben-Zeev, 2014). Participants were asked EMA questions throughout the day regarding positive affect (“How excited do you feel right now”) and negative affect (“How upset do you feel right now?”) (Brenner & Ben-Zeev, 2014). The expectation of emotionally charged days could be attributed to prior experiences, both positive and negative, and the need to mentally prepare oneself for that. The ability to tolerate distressing things could be useful when thinking about negative experiences, and the desire to not feel such strong negative emotions.

When considering potential moderators of affective forecasting, distress tolerance may be one potential candidate. Distress tolerance can be defined as the capacity to experience and withstand psychological states. It consists of an individual’s expectations and evaluations of experiencing negative emotional states in respect to four domains: (1) tolerability and aversiveness, (2) appraisal and acceptability, (3) tendency to absorb attention and disrupt functioning, and (4) regulation of emotions (Simons & Gaher, 2005). Examples of distress tolerance skills can include deep breathing exercises and mindful observation of thoughts. Increasing levels of distress tolerance is important in individuals given prior work highlighting increased levels of anxiety and depression in those who endorse lower distress tolerance (Li et al., 2023).

The connection between distress tolerance and affective forecasting shows that individuals with lower distress tolerance predicts use of maladaptive emotion regulation strategies such as rumination, avoidance, and suppression of feelings (Brown et al., 2022). Someone with low distress tolerance might overestimate how negative a situation will make them feel and could lead to them avoiding potentially beneficial experiences due to the fear of discomfort (Larrazabal et al., 2022). Relating back to affective forecasting, this may result in individuals who do not tolerate distress well end up more likely to experience negative emotion on days when they expect to have a less positive day. This could be due to possibly generating negative events and negative emotion because they expect the day to be bad. Indeed, this idea has been studied in related areas, such as stress generation (Liu & Alloy, 2010; Liu et al., 2024). It could also be that those with better distress tolerance are better able to navigate the negative events they anticipate experiencing and thus see less of an emotional impact from the events. The model we propose here is best measured using methods that can capture affect as it occurs, one such method for doing so is ecological momentary assessment (EMA). We hypothesized that with distress tolerance as a moderator, distress tolerance will strengthen the relationship between EMA daily ratings of the day and negative affect.

## Method

### Participants

This study utilized data from a sample of college students that participated in a larger ecological momentary assessment (EMA) study (*N* = 675). Students had to be at least 18 years old to participate in the study and have access to a compatible iOS or Android smartphone. We included participants in this subsample who completed baseline data and at least three EMA surveys. This resulted in a total of 418 participants.

### Procedures

The data were used from a study in which information was collected at baseline and from an EMA period up to six times a day. Surveys were sent at random times to participants during the day, for eight weeks. Surveys were sent within a pre-defined period (typically 14 hours) at random intervals within windows of at least 2 hours. The 414 participants completed a total of 42,578 surveys (mean = 102.85 each, *SD* = 109.11). Participants completed at least one survey per day for 32.17 days on average (*M* = 19.71 days).

### Measures

#### Distress Tolerance Scale

The Distress Tolerance Scale (DTS) is a 15 item self-report measure of emotional distress tolerance (Simons & Gaher, 2005). This measure asked participants “Think of times that you feel distressed or upset. Select the item that best describes your beliefs about feeling distressed or upset.” Participants were asked 15 statements including “I keep my emotions to myself” and “I’ll do anything to stop feeling distressed or upset” and ranked their responses on a 1–5 Likert scale (Strongly Agree, Mildly Agree, Feel Neutral, Mildly Disagree, Strongly Disagree). The Distress Tolerance Scale has been shown to have good reliability and ability, demonstrating strong consistent internal consistency in prior work with Cronbach’s alpha ecoefficiencies typically ranging, from .80 to .90, displaying strong convergent and discriminant validity across various studies and populations, indicating it effectively measures its intended construct of distress tolerance **(**Oorjitham et al., 2024).

#### EMA Assessments

Each morning, participants were asked “How much do you believe this statement: Today will be a good day.” Participants ranked this from 0 to 100%. Throughout the day, participants were asked a series of affect questions stated as, “Right now, how much do you feel:” on a 1–10 scale assessing: desire to escape, sad, stressed, hopeless, down on self, anxious, worried, agitated, and lonely.

We created a composite variable consisting of all affect states, at each time point. We then created a day-level average of the negative affect ratings for all assessments after the initial morning survey. Negative affect items were selected for this analysis based on all variables in the overall study that participants could select and choosing options that reflected feelings of emotional distress or unhappiness, including “sad,” “hopeless,” and “worried” (Mirams et al., 2014).

### Analytic Strategy

We conducted a multi-level model (days within people) with a cross-level interaction between daily ratings (person-mean centered) of how good the day would be and distress tolerance. We included the person-mean of daily ratings of how good the day would be to tease apart between- vs. within-person effects. All analyses were done using the lme4 R package. Follow-up moderation probes and plots were conducted with the interaction and sJplot packages.

## Results

Our sample had an average age of 19.44 years old (*SD* = 1.88), and additionally, our sample identified as predominantly Asian (48%) and White (35%); however, we had multiple participants identify as Black (6%), Other/Multiple races (10%), as well as American Indian/Alaskan Native (1%). Gender also varied within our sample; while our population was mainly Female (71%), there was a considerable number of Male participants (26%), as well as individuals who identified as non-binary/gender non-conforming (1%), transgender male (1%), and gender not-listed (1%).

**Fig. 1:**
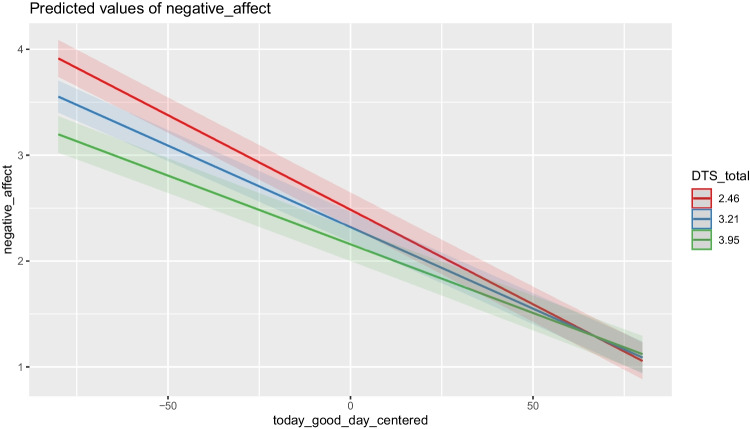
Moderation graph. This figure displays the moderation graph showing the interaction between negative affect, distress tolerance, and daily ratings of “Today will be a good day”

As can be seen in Table 1, there was an overall negative effect where lower ratings of how good people thought the day would be were associated with more negative affect later in the day. Distress tolerance moderated this relationship such that the relationship between ratings of the day and negative affect was stronger for those who had below average distress tolerance (− 1*SD*; *b* = − 0.018, *t* = − 44.725, *p* < .001) compared to those with average (*b* = − 0.015, *t* = − 50.566, *p* < .001) and above average (+ 1*SD*; *b* = − 0.013, *t* = − 29.62, *p* < .001) distress tolerance. See Fig. 1 for a visualization of this relationship.

**Table 1:** Moderation analyses table

Predictors	Estimates	CI	*p*
(Intercept)	5.0865	4.6124–5.5606	**< .001**
Person-level average of daily ratings	− 0.0362	− 0.0429 to − 0.0296	**< .001**
Daily rating (person-mean centered)	− 0.0259	− 0.0284 to − 0.0235	** < .001**
DTS total	− 0.1924	− 0.2827 to − 0.0998	** < .001**
DTS × daily rating	0.0033	0.0025–0.0040	** < .001**
Random effects			
*σ*^2^	0.91		
τ_00__ID_	1.68		
ICC	0.65		
N _ID_	414		
Observations	42,578		
Marginal *R*^2^ /conditional *R*^2^	0.180/0.712		

Moderation Analyses Table. Moderation analyses showing interactions between negative affect, distress tolerance and daily ratings of “Today will be a good day”. Values in bold indicate significance

## Discussion

The goal of this study was to examine distress tolerance as a moderator of the relationship between daily forecasts of how good a day would be and the resulting momentary negative affect experienced later in the day. We reasoned that poorer distress tolerance would lead to a stronger (negative) relationship between daily ratings of how good a day would be and negative affect later in the day. Our hypothesis was supported: we found that those with poorer distress tolerance, there is a stronger negative relationship between daily ratings of how good a day would be and negative affect later in the day compared to those with better distress tolerance skills. This research highlights distress tolerance as a moderator of affective forecasting, and shows where future studies can expand upon this work.

One interpretation of these findings is that individuals who have less optimism regarding how well a day goes are likely to experience more negative affect despite being exposed to distressing things. As highlighted in Steindl et al. (2021), an individual’s ability to deal with their own suffering can lead to distress, which could potentially lead someone to experiencing more negative affect. Additionally, the ability to deal with distressing things can play a role in overall optimism and affect. Individuals who have difficulty in dealing with distressing events may have less belief in how well a day would go and thus experience negative affect later into the day. These findings indicate an expectation effect, indicating individuals’ beliefs or expectations about an event influencing actions and ultimately causing the outcome to occur (Madon et al., 2011). Specifically, participants were anticipating the day would go poorly, were not equipped with distress tolerance skills, and thus experienced negative affect later in the day.

A broader implication of these findings highlights how practicing distress tolerance can in turn promote emotional resilience. Distress tolerance includes the ability to actively manage intense emotions rather than avoid, and by building distress tolerance skills, individuals can develop tools to cope in stressful situations, which in turn promotes resilience. Especially in college students, the ability to tolerate distress shows higher degrees of cognitive flexibility (Arici-Ozcan et al., 2019). Another aspect of distress tolerance relevant to the findings is that the ability to accept a situation without judgment would be beneficial especially within instances where individuals predict the day will be bad before it has happened. Acquiring tools to be able to accept reality as it is, without judgment towards it would build distress tolerance and likely decrease negative affective forecasting.

There are some limitations/future explorations within this study to note as well. Our sample was predominantly White and Asian and so future studies should look to replicate with a more naturally representative sample of college students. Other limitations include age given our sample only included college students, as well as education level given the sample. Future research should look to expand upon this work beyond college students, including adolescents as well as older adults to examine whether there are differences in affective forecasting. Future work should also look to examine distress tolerance as a behavioral task compared to questionnaire form. Distress tolerance was conceptualized as the perception of one’s ability to tolerate distress rather than direct ability in our study, and future work can expand upon this by examining direct ability. Additionally, future research should look to further explore the relationship that exists between daily ratings of how good a day would be and predicting affect. As researchers continue to use EMA to examine individuals on a day-to-day basis, more research focused on experience of emotion in connection with how individuals believe a day will pan out for them should be considered. Future exploration of this could allow for more research examining the connection and how it relates to individuals with emotionally charged weeks and those who experience poor distress tolerance.

Despite these limitations, the present study has enhanced our understanding of the relationship between daily ratings of how good a day would be and negative affect, with distress tolerance as a moderator. We hope that this current study will encourage further investigation of how distress tolerance moderates the relationship between daily ratings of the day and affective forecasting, especially in larger community samples.

## Additional Information

### Data Availability

Data that supports the findings of this study have been deposited in Open Science Framework, with the identifier here:  10.17605/OSF.IO/U8QSW.
